# Factors associated with leakage in patients with an ostomy: A cross‐sectional study

**DOI:** 10.1002/nop2.1612

**Published:** 2023-01-24

**Authors:** Kirsten Lerum Indrebø, Anny Aasprang, Torill Elin Olsen, John Roger Andersen

**Affiliations:** ^1^ Department of Surgery Førde Central Hospital Førde Norway; ^2^ Faculty of Health and Social Sciences Western Norway University of Applied Sciences Førde Norway; ^3^ Centre of Health Research Førde Hospital Trust Førde Norway; ^4^ Faculty of Health and Social Sciences Western Norway University of Applied Sciences Bergen Norway; ^5^ Department of Surgery Haukeland University Hospital Bergen Norway

**Keywords:** colostomy, clinical feedback system, education, ileostomy, leakage, ostomy, outpatient follow‐up, patient‐reported outcomes, stoma care nurse, urostomy

## Abstract

**Aims:**

To explore the associations between sociodemographic and clinical data, the patient's knowledge and skills, and relationship to healthcare professionals with leakage from an ostomy.

**Design:**

Cross‐sectional.

**Methods:**

This study included 160 patients with a colostomy, ileostomy, or urostomy. Leakage was the dependent variable and was assessed by self‐report. Sociodemographic and clinical data and the Ostomy Adjustment Scale subscores, ‘knowledge and skills’ and ‘health care professionals’ were independent variables. Spearman's rho and multivariate partial least squares regression analysis were used to estimate possible factors associated with leakage.

**Results:**

Of the participants, 13.8% had leakage weekly or more often, 16.3% more often than once a month and 37, 5% had leakage more seldom than once a month. The most important risk factors for leakage were (1) having an ostomy placement that does not meet international guidelines, (2) not having an optimal relationship with health professionals, (3) having a diagnosis other than cancer, (4) not having proper knowledge and skills in ostomy care, (5) not having a colostomy, (6) having a convex baseplate, (7) having an oval ostomy, and (8) being dependent on others for ostomy care. The independent variables in the PLS‐ model explained 31% of the variance in leakage.

**Patient or Public Contribution:**

We thank the patients in the user panel for their help during the study.

## 1 INTRODUCTION

In Norway, about 20,000 people live with an ostomy (Krabbe et al., 2019). The main reasons for ostomy surgery are cancer or inflammatory bowel disease (IBD). Still, several other issues may also result in an ostomy, such as incontinence, infections, constipation, or complications from surgery (Ambe et al., 2018; Olsen et al., 2020). Faeces, gas, or urine exit the intestine through a surgically created opening on the abdomen (ostomy). The waste enters an external bag, which is part of the ostomy equipment. This equipment includes a skin barrier, which adheres to the abdominal skin and a collecting bag. The equipment can be in one or two pieces—skin barrier and pouch. The bag must be emptied or changed from one to several times each day, and the skin barrier must be changed daily or less often depending on the type of equipment and the ostomy type.

The equipment may not always be perfectly fitted to the ostomy and the skin surrounding it, resulting in leakage. Leakage can be defined as waste under the ostomy skin barrier in contact with skin. Still, patients may also define leakage when waste first appears outside the skin barrier into the clothes. Leakage is reported to happen in 17%–87% of patients (Bulkley et al., 2018; Claessens et al., 2015; Cruz et al., 2021; Feddern et al., 2015; Haugen et al., 2006; Maydick, 2016; Pearson et al., 2020; Pittman et al., 2008; Porrett et al., 2011; Ratliff, 2014; Rutherford et al., 2020), and it seems to persist over time (Pearson et al., 2020). Thus, it is a well‐known challenge for patients following ostomy surgery, both the short‐ and long‐term, and it affects patients' everyday life physically, psychosocially, and socially (Haugen et al., 2006; Rutherford et al., 2020; Sun et al., 2020).

Leakage is one of several complications after ostomy surgery, and quantitative studies have reported that women have four times more frequent leakage than men and that having one‐piece ostomy equipment was associated with more frequent leakage (Ratliff, 2014). Other reasons for leakage include not having the ostomy sited preoperatively (Cruz et al., 2021), having a low or retracted ostomy (Lindholm et al., 2013), having an abnormal abdominal contour and overfilled ostomy bag (Haugen et al., 2006), and reduced visibility of the skin around the ostomy (McMullen et al., 2011).

Qualitative studies have identified that not having well‐fitting equipment, peristomal skin irritation or allergic contact dermatitis, or an ileostomy or urostomy are risk factors for leakage (Sun et al., 2020). Other qualitative studies have reported that patients felt unprepared to handle the situation when leakage happened after leaving the hospital (Werth et al., 2014). Patients wanted more professional help with ostomy care (Cengiz & Bahar, 2017), but because patients felt guilty when leakage appeared, they did not always seek help from healthcare professionals (Rutherford et al., 2020).

The patient must report leakage, and it is, therefore, essential for the patient to understand what leakage is. The patient's perceived knowledge about observations of ostomy, skin, and practical care is not generally reported as a factor associated with leakage in the studies mentioned above. Nevertheless, several studies have shown that leakage from ostomy equipment occurred frequently and persisted for several years after surgery, negatively affecting the patient's adjustment to life with an ostomy and quality of life (Bulkley et al., 2018; Haugen et al., 2006; Parmar et al., 2011; Pittman et al., 2008; Ratliff, 2014; Rutherford et al., 2020; Sun et al., 2020).

Leakage can also cause several skin problems, such as chemical dermatitis, maceration, candida, crystallization, and pseudoverrucous lesions. Altogether, leakage problems are challenging for many patients, and identifying individual reasons for leakage is the first step towards prevention.

Consequently, research is needed that explores the effects of factors on leakage. This study investigated some of the previously mentioned factors associated with leakage and aimed to identify any new factors. Thus, the purpose of this study was to explore the associations between sociodemographic factors, clinical factors, the patient's knowledge and skills, and relationship to health professionals, with leakage, in patients in a follow‐up program by stoma care nurses (SCN).

## 2 MATERIALS AND METHODS

This cross‐sectional study was conducted in the outpatient ostomy clinic in Førde Central Hospital from 2018–2021. The patients had a colostomy, ileostomy, or urostomy, and they were followed up by SCN regularly at 3 weeks, 3, 6, and 12 months postoperatively and after that annually. At the data collection time, patients had had an ostomy of either 3, 6, or 12 months, or between 1–15 years.

### 2.1 Inclusion

The inclusion criteria were patients aged ≥18 years old, had a colostomy, ileostomy, or urostomy for ≥3 months, and could speak, read, and write Norwegian.

### 2.2 Procedure

Three SCNs educated in accordance with the World Council of Enterostomal Therapists (WCET) recommendations followed up the patients in an outpatient clinic with the use of electronically patient‐reported outcomes (PROs) and a clinical feedback system (CFS) (Indrebø et al., 2020). The follow‐up was conducted in accordance with the national recommendations for follow‐up of ostomy patients in Norway (Olsen et al., 2020). Before each consultation, the patients responded to electronic questionnaires, including sociodemographic and clinical items and the Ostomy Adjustment Scale (OAS). In each follow‐up consultation, an SCN conducted a clinical observation of the ostomy, the skin, and the ostomy equipment. The findings and whether new measures were needed were discussed with the patient.

### 2.3 Dependent variable

The reported frequency of leakage by patients was the dependent variable, and leakage was recorded into five categories: ‘never’, ‘less than once a month’, ‘more than once a month’, ‘weekly’, or ‘several times a week’. There is no consensus among SCNs of the definition of leakage. In this study, leakage was defined as waste exposed to the skin under the ostomy skin barrier. The fact that leakage may occasionally happen and, therefore, never be entirely absent was a difficult factor to consider. Nevertheless, it was decided to include ‘never having leakage’ as an answer category.

### 2.4 Independent variables

#### 2.4.1 Sociodemographic and clinical form answered by patients

The form included items about the patients' age, marital status, education, frequency of leakage from the ostomy equipment, vision and hand function, and “degree of ostomy self‐care”. The variables were recorded as binary scores; gender was either male or female; marital status was either married/cohabitant or living alone; education was <13 years (low) or >13 years (high). Clinical data were scored as having or not having reduced hand/finger function or reduced vision. Self‐care of the ostomy was recorded as independent or needing help in daily ostomy care (Indrebø et al., 2014).

#### 2.4.2 Knowledge and skills and health professionals

The OAS with seven subscales has been validated in the Norwegian population. The model fit of the OAS was tested with robust confirmatory factor analysis and produced acceptable results. The composite reliability value of the knowledge and skills subscale was 0.80, and that of the health professionals subscale was 0.78 (Indrebø et al., 2021). The subscale knowledge and skills included two items. One item related to whether the patient felt well educated in ostomy care and whether the patient was confident that they knew the correct methods for managing the ostomy. The subscale health professionals included three items: two items about whether the patient discussed even the most embarrassing aspects of the ostomy with the doctor, and whether the patient avoided discussing changes in the ostomy; and one item was about whether the patient felt like a ‘complainer’ when they contacted the doctor or SCN (Indrebø et al., 2021). ‘The patients responded to the OAS on a LIKERT scale ranging from 1‐6, where higher scores indicated better adjustment’.

#### 2.4.3 Clinical form answered by SCNs

The form included time since surgery, diagnosis, ostomy type and shape, parastomal hernia, parastomal skin condition, ostomy equipment, and ostomy site. The cancer group included colorectal, bladder, prostate, and ovarian cancer. Patients who had radiochemotherapy were also included. The ‘other’ group consisted of patients with diverticulosis, faecal incontinence, constipation, and complications after radiation therapy. BMI was calculated with the standardized formula (World Health Organization, 2019).

Ostomy shape was measured with callipers; oval‐shaped was described as a >3 mm difference in the diameter of the ostomy. The ostomy length and height from parastomal skin level to the top of the ostomy were measured when the patient was lying down. There is no consensus among surgeons and SCNs about an optimal ostomy length for the three types of ostomy. Still, a study from the U.K found that an insufficient stoma height of <10 mm was associated with more frequent ostomy complications (Cottam et al., 2007).

The parastomal hernia was defined as a defect in the abdominal fascia that allows the intestine to bulge into the parastomal area (Colwell & Beitz, 2007). It was measured as a palpable protrusion or bulge adjacent to the stoma. The surgeon provided a second opinion if the SCN was unsure whether it was a parastomal hernia.

Peristomal dermatitis included peristomal irritant contact dermatitis, allergic contact dermatitis, and peristomal moisture‐associated dermatitis (MASD). MASD causes peristomal erosion due to damage from skin exposure to faecal or urinary drainage, chemical preparations, or an inflammatory skin response resulting from hypersensitivity to chemical elements or loss of peristomal epidermis (Colwell et al., 2011; Colwell & Beitz, 2007).

Dermatitis was measured with clinical judgement by SCNs. The ‘other skin conditions’ included skin complications, such as peristomal varices, candidiasis, folliculitis, mucosal transplantation, pseudoverrucous lesions, and peristomal granulomas, as defined by Colwell and Beitz (2007).

Ostomy equipment was defined as pouches used to collect waste from the ostomy. Both one‐ and two‐piece pouches in several sizes were included. The skin barriers were divided into flat or convex skin barriers. All convex skin barriers, both soft and hard/deep convexes, were included. Preoperative ostomy siting and postoperative ostomy placement were judged against whether they aligned with national and international guidelines. The American Society of Colon and Rectal Surgeons Committee Members, & Wound Ostomy Continence Nurses Society Committee Members (2007) claims that ostomy should be cited in the rectus muscle, away from scars, navel, folds, and bone protrusions, well visible and accessible so the patient can take care of it themself. It should be checked that the criteria are met when the patient is lying, sitting, standing, bending forward, and to both sides (Alstad, 1997; “American Society of Colon and Rectal Surgeons Committee Members, & Wound Ostomy Continence Nurses Society Committee Members,” 2007).

Elective patients received information before admittance to the hospital for surgery. The information was in accordance with the Nordic recommendations for preoperative information (Alstad, 1997). The information included why and how the ostomy should be constructed, expected consequences for everyday life were discussed, the patient was shown ostomy equipment, and the stoma localization was examined. The patients had no practical training before surgery. The SCN had at least one patient consultation postoperatively before discharge from the hospital. The variables were recorded as binary scores: ‘having’ or ‘having not’; cancer, IBD, other diseases or conditions; colostomy, ileostomy, urostomy, two ostomies (colostomy and urostomy); preoperative ostomy site marked, ostomy site marked by an SCN, ostomy placed according to the international guidelines, and parastomal hernia/bulge. Ostomy equipment was recorded as two pieces (skin barrier and pouch) or one piece. The skin barrier was recorded as flat or convex, and the ostomy's shape was recorded as circular or oval. Time since surgery was recorded as <1 year or >1 year since the operation. The length of the ostomy was recorded into five categories: flush in or below skin level, 0.1–2 cm over skin level, 2.1–4 cm over skin level, 4–6 cm over skin level, or >6 cm over skin level. Age and BMI were recorded as continuous variables.

### 2.5 Statistics

The characteristics of the sample (*n* = 160) are presented as numbers and percentages or means ± standard deviations. To assess the unadjusted correlations between leakage and the independent variables, Spearman's rank‐order correlation (Spearman's *rho*) was used because this analysis method is designed to handle ordinal variables (Altman, 1990; Pallant, 2016). Two‐tailed *p*‐values were reported. Furthermore, a multivariate partial least squares (PLS) analysis was conducted to explore which variables were significant factors for leakage. PLS regression analyses ordinal dependent variables and decomposes the explanatory variables into orthogonal linear combinations (PLS components) while simultaneously maximizing the covariance with the outcome variable. PLS regression also allows for multivariate modelling in samples with many variables related to observations (Wold et al., 1984). All variables were standardized, and then selectivity ratios (SRs) with 95% confidence intervals were calculated, defined as the ratio of the explained predictive variance to the total variance for each independent variable. The SR‐values ranged from −1 to 1. The model was cross‐validated using Monte Carlo resampling with 1000 repetitions repeatedly and randomly keeping one‐quarter of the patients as an external validation sample (Rajalahti, Arneberg, Berven, et al., 2009; Rajalahti, Arneberg, Kroksveen, et al., 2009). Also, the total explained variance of the model on leakage was reported. The PLS analysis was performed using Sirius vs. 11.0 (Pattern registration systems, Bergen, Norway), while other analyses were conducted using SPSS version 24.

### 2.6 Ethics

In accordance with the Helsinki Declaration, the study was reviewed and approved by the Regional Committee for Medical Research Ethics in Western Norway (registration number 2016/255).

## 3 RESULTS

The sample consisted of 160 ostomy patients (ileostomy, *n* = 56), (colostomy, *n* = 75), (urostomy *n* = 17), and colostomy and urostomy (*n* = 12). Missing data were rare; three participants (1.9%) for the variable ‘hand function’ and two (1%) for the variable ‘preoperative information before hospitalization’. For the OAS subscores ‘knowledge and skills’ missing was one (0.6%), and for ‘health professionals’ it was seven (2.3%). Patient characteristics and leakage frequency are presented in Table 1.

**TABLE 1:** Sample characteristics and frequencies of leakage

Variables	*N* (%)
Leakage	
Never	52 (32.5)
Less often than once a month	60 (37.5)
More often than once a month	26 (16.3)
Weekly	12 (7.5)
Several times every week	10 (6.3)
Gender	
Woman	69 (43.1)
Men	91 (56.9)
Age (years) mean (SD)	64 (15.3)
Education	
<12 years	120 (75)
>12 years	40 (25)
Marital	
Married/cohabitant	110 (68.8)
Living alone	50 (31.3)
Self‐care of the ostomy	
Yes	132 (82.5)
No	28 (17.5)
Vision	
Normal	72 (45)
Reduced	88(55)
Reduced hand/finger function	
No	112 (70)
Yes	45 (21.1)
BMI mean (SD)	24.8 (3.6)
Time since surgery	
<1 year	45 (28.1)
>1 year or more	115 (71.3)
Type ostomy	
Ileostomy	56 (35)
Colostomy	75 (46.9)
Urostomy	17 (10.6)
Two ostomies (colo and uro)	12 (7.5)
Diagnosis	
Cancer	86 (53.8)
IBD (inflammatory bowel disease)	42 (26.3)
Other diseases or conditions	32 (20)
Preoperative information before hospitalization	
Yes	104 (65)
No	54 (34)
Preoperative site marking according to international guidelines	
Yes	130 (81.3)
No	30 (18.8)
Postoperative ostomy placement according to international guidelines	
Yes	132 (82.5)
No	28 (17.5)
Ostomy site marked by SCN	
Yes	102
No	58
Ostomy length	
Flush to or below skin level	20 (12.5)
0, 1–2 cm over skin level	55 (34.4)
2, 1–4 cm over skin level	78 (48.8)
4, 1–6 cm over skin level	6 (3.8)
>6 cm over skin level	1 (0.6)
Ostomy shape	
Circular	86 (53.8)
Oval‐shaped	74 (46.3)
Parastomal hernia/bulge	
No	131 (81.9)
Yes	29 (18.1)
Ostomy equipment	
Two‐piece (skin barrier and pouch)	122 (76.3)
One piece	38 (23.8)
Skin barrier	
Flat	113 (70.6)
Convexity (all types)	47 (29.4)
Skin	
Normal	84 (52.2)
Rubor	46 (28.7)
Dermatitis^a^	9 (5.6)
Other skin disorders^b^	21 (13.1)
Knowledge and skills (OAS subscore) mean (SD)	5.4 (0.9)
Health professionals (OAS subscore) mean (SD)	5.3 (0.1)

^a^ Irritant, allergic, or parastomal trauma.

^b^ Psoriasis, candida, crystallization, granulomas, peristomal varices, candidiasis, folliculitis, mucosal transplantation, and pseudoverrucous lesions.

The independent variables in the PLS‐model explained 31% of the variance in leakage. The most influential statistically significant risk factors for leakage were (1) not having an ostomy placement according to international guidelines, (2) not having an optimal relationship with health professionals, (3) having an ‘other’ diagnosis than cancer, (4) not having proper knowledge and skills in ostomy care, (5) not having a colostomy, (6) having a convex baseplate, (7) having an oval ostomy, and (8) being dependent on others for ostomy care (Table 3).

In the correlation analysis (Table 2), the factors mentioned above were significantly correlated with leakage. In addition, higher BMI scores, time since surgery, and the use of convex skin barrier were significantly correlated with the frequency of leakage.

**TABLE 2:** Correlations between leakage and sociodemographic variables, clinical variables, and the Ostomy adjustment subscores knowledge and skills (KS) and health professionals (HP)

Variables	Spearman's *rho*
Ostomy placement according to international guidelines (yes = 0, no = 1)	0.237**
Skin barrier (flat = 0, convex = 1)	0.177*
BMI (continuous)	0.174*
Time since surgery (<1 year = 1, >1 year or more = 2)	0.163*
Other diseases or conditions (no = 0, yes = 1)	0.162*
Ostomy shape (round = 0, oval = 1)	0.157*
Self‐care of the ostomy (yes = 0, no = 1)	0.142
Gender (women = 0, men = 1)	0.125
Marital (married/cohabitant = 0, living alone = 1)	0.138
Urostomy (no = 0, yes = 1)	0.111
Ostomy equipment (two‐piece [skin barrier and pouch] = 0, one‐piece = 1)	0.107
Ostomy site marked by SCN (yes = 0, no = 1)	0.092
IBD (inflammatory bowel disease) (no = 0, yes = 1)	0.092
Two ostomies (colo and uro) (no = 0, yes = 1)	0.091
Ileostomy (no = 0, yes = 1)	0.082
Preoperative information before hospitalization (no = 0, yes = 1)	0.045
Preoperative site marking according to international guidelines (yes = 0, no = 1)	0.032
Sight (Normal = 0, reduced = 1)	0.023
Education, (>12 years = 0, <12 years = 1)	0.001
Reduced hand/finger function (no = 0, yes = 1)	−0.005
Parastomal hernia/bulge (no = 0, yes = 1)	−0.059
Ostomy length flush to or below skin level= 0, 0.1–2 cm over skin level = 1, 2.1–4 cm over skin level = 2, 4–6 cm over skin level = 3, or >6 cm over skin level = 4	−0.044
Age, years (continuous)	−0.133
Colostomy (no = 0, yes = 1)	−0.195*
Cancer (no = 0, yes = 1)	−0.211**
Health professionals (continuous)	−0.258**
Knowledge and skills (continuous)	−0.337**

*Note*: Positive values indicate that the variables are associated with more leakage and vice versa.

* *p* ≤ 0.05

** *p* ≤ 0.001.

## 4 DISCUSSION

This study identified both changeable and unchangeable factors associated with leakage frequency. Factors such as knowledge and skills and the relationship with health professionals are possible changeable factors. Optimal ostomy placement is an unchangeable factor for patients who have already had surgery, but practice can be improved for future patients. Self‐care for the ostomy can be improved in some patients, for example, by learning new skills or optimizing the equipment. The reason for surgery, such as other diagnoses than cancer, and the shape of the ostomy is unchangeable, but the patient must consider it in practical ostomy care. Although some of the findings in the study confirm those in previous studies (Braumann et al., 2019; Bulkley et al., 2018; Cannon & Hayden, 2019; Colwell & Gray, 2007; Cottam et al., 2007; Cruz et al., 2021; Feddern et al., 2015; Haugen et al., 2006; Herlufsen et al., 2006; Kim et al., 2021; Millan et al., 2009; Pearson et al., 2020; Person et al., 2012; Pittman et al., 2008; Ratliff, 2014; Redmond et al., 2009; Sun et al., 2020; Williams et al., 2010), direct comparisons must be viewed with caution. For example, there are different designs and methods for data collection and inclusion criteria. The patients have other diagnoses, definitions on leakage are unclear, and the frequency of leakage is often not directly comparable.

### 4.1 The findings in relation to the literature

#### 4.1.1 Leakage frequency

The overall leakage frequencies in the study were mostly the same as in previous literature, suggesting leakage is a problem for many ostomy patients. Cross‐sectional studies have reported leakage frequencies from 61%–87%, and a frequency of monthly leakages from about 10%–36%, and weekly leakage frequency from 6%–32% (Feddern et al., 2015; Ratliff, 2014; Williams et al., 2010). A qualitative study found that leakage was the most frequently reported challenge, regardless of time, after ostomy surgery (Sun et al., 2020). In a longitudinal study among patients with faecal ostomies with 2 years of SCN follow‐up, the leakage frequency was 87%. In a substantial proportion of patients, leakage frequency increased over 2 years, particularly night‐time leakage in patients with ileostomies. The study discussed the importance of closer cooperation between patients and SCN over the longer term (Pearson et al., 2020).

#### 4.1.2 Ostomy placement according to international guidelines

Results showed that leakage from ostomy was associated with inappropriate ostomy placement, which is also supported by other studies (Colwell & Gray, 2007; Kim et al., 2021; Millan et al., 2009; Person et al., 2012). In several studies, the frequency of preoperative site marking differs from nearly all ostomies to less than half. Patients who are operated on as an emergency less often have the ostomy site marked preoperatively than those with elective surgery. Those patients are more likely to have leakage and skin problems (Cruz et al., 2021; Millan et al., 2009; Pearson et al., 2020; Person et al., 2012). This study did not differ between acute and elective surgery, but >80% of the patients had an ostomy placement in line with international guidelines. Although the study showed a high frequency of stoma site marking, 67.5% of the patients experienced leakage, indicating there are also other reasons for leakage than the ostomy placement.

#### 4.1.3 Leakage and relationship to health professionals

The results showed a significant association between having a good relationship with a health professional and a lower frequency of leakage. Several studies have confirmed that patients who felt confident to contact and discuss all aspects of the ostomy with the SCN and doctor adjusted better to live with an ostomy (Cannon & Hayden, 2019; Cottam et al., 2007; Cruz et al., 2021; Haugen et al., 2006) compared with those who did not feel confident to make contact (Bulkley et al., 2018; Redmond et al., 2009). The reason for not making contact could be that the patient felt guilty and thought leakage was something the patient had to cope with alone, or there was no SCN available (Rutherford et al., 2020). The findings in the study could be explained because the patients had a clinical follow‐up, and, in addition, they had the opportunity for telephone or drop‐in consultations as needed.

#### 4.1.4 Leakage and knowledge and skills

Lower risk of leakage was found in patients with good knowledge and skills. However, some patients did not understand that they had leakage under the skin barrier and had answered that they did not experience leakage on the PRO questionnaire. In two studies about skin complications, some patients did not identify skin problems themselves, and the findings were explained with lack of education and follow‐up by SCN (Herlufsen et al., 2006; Williams et al., 2010). In a cross‐sectional study, patients ranked practical skills as the most important learning theme in the short term after surgery. Several patients felt unprepared when the first leakage appeared after discharge from the hospital (Werth et al., 2014). A meta‐synthesis of qualitative studies described, for example, that patients wanted tailored information about dietary advice, which could have an impact on output consistency and leakage frequency for some patients (Rutherford et al., 2020).

In this study, preoperative information about the ostomy was provided in an outpatient clinic the same day as the doctor and patient agreed on surgery. There was only a short time for practical education in this situation, and perhaps not the most helpful timing. The government's goal is that waiting times, especially for cancer treatment, should be as short as possible (Helsedirektoratet). Patients who do not need radiotherapy or chemotherapy should have a short waiting time from diagnosis to operation. Ostomy patients may also be included in the enhanced rehabilitation after surgery (ERAS) program (Forsmo et al., 2016). Both those factors may reduce the time available for education in practical ostomy care both pre‐and peri‐operatively.

#### 4.1.5 Leakage and diagnosis

The study showed that people with cancer had fewer severe leakage problems than participants who underwent ostomy surgery due to other diagnoses. These findings are consistent with Pittman et al. (2008) and Cottam et al. (2007). An explanation may be that many of the people with cancer had an end colostomy, and those patients have been found to have less leakage compared with others (Ratliff, 2014).

#### 4.1.6 Leakage and ostomy type

Patients with a colostomy had less leakage than patients with other ostomy types in this study. Parmar et al. (2011) found that patients with a colostomy experienced more skin problems than those with an ileostomy, but other literature has demonstrated contradictory results (Ratliff, 2014). Conflicting results may be explained by sample variation, for example, the numbers of patients with a loop colostomy, such as in the study by Parmar et al. (2011), or an end colostomy, such as in this research. In Norway, loop ileostomies are usually preferred instead of transverse or loop colostomies to protect anastomosis in rectal cancer surgery. In this study, patients were excluded after having a loop‐ileostomy for <3 months.

#### 4.1.7 Leakage and type of baseplate

This study also found associations between the type of skin barrier and leakage. The need to change from a flat to a convex baseplate was a ‘marker’ because the patient had leakage problems. A convex skin barrier could prevent leakages in low ostomies, especially if its shape, depth, and rigidity fit precisely the ostomy and surrounding skin (Hoeflok et al., 2017). In this study, one of the three patients needed to change from a flat to a convex skin barrier, which reduced the leakage frequency. Thus, changing from a flat baseplate to convexity seems to be a good treatment for leakage in certain circumstances. Stoma height is one indication of the use of a convex skin barrier. Still, several other factors, such as stoma opening in or below skin level, liquid output, bulges surrounding the ostomy, and body figure also can be indicators (Hoeflok et al., 2017; Wound, Ostomy and Continence Nurses Society et al., 2015). In this study, only 12.5% of ostomies were flush to or below skin level; in 34.4%, the ostomy was 0.1–2 cm over skin level. The positive effect of convexity on leakage may be explained by regular follow‐up and careful adjustments in ostomy equipment, which also is discussed in another study (Carlsson et al., 2016). A study found that convexity was more frequently used in patients having emergency operations than in planned operations, 46% versus 25% of the participants (Carlsson et al., 2016). Still, this study did not differ between emergency and planned surgery.

#### 4.1.8 Leakage and ostomy shape and length

The study showed associations between leakage and an oval ostomy shape, contradicting Carlson et al. (Carlsson et al., 2016). A cause of the leakage frequency in patients with an oval ostomy could be that a skin barrier with the circular pre‐cut opening was used more frequently in this study compared with the study by Carlson et al. (2016); no statistically significant associations between leakage and ostomy length were found in this study. Lindholm et al.'s (2013) study among 144 faecal ostomy patients found that ostomy length became shorter during the first weeks postoperatively, and an oval ostomy shape was most frequent in the first 6 months postoperatively Lindholm et al. (2013). Studies showed that risk factors for leakage were both the type of ostomy, such as loop ostomies and stoma height <10–5 mm (Cottam et al., 2007).

#### 4.1.9 Leakage and independence with ostomy care

The results found that participants dependent on others in ostomy care had more leakages than participants who were independent of others (Table 3). Qualitative studies have described obstacles in managing practical ostomy care, such as difficulties in seeing the ostomy and the surrounding skin and applying the ostomy wafer to the skin (McMullen et al., 2011; Sun et al., 2020). Patients who received help from partners had fewer leakages (McMullen et al., 2011). In those studies, the education and follow‐up programs were not described. Bulkley et al. (2018) studied self‐care problems among 177 rectal cancer survivors ≥5 years post‐diagnosis, and approximately 25% reported leakage and skin problems as the most common self‐care problem. Of the participants, 22% reported difficulty caring for the ostomy (Bulkley et al., 2018). Contradictory to the studies mentioned above, a systematic outpatient follow‐up was conducted in this study, and 82.5% of patients were self‐sufficient in ostomy care.

**TABLE 3:** Partial least square regression prediction analysis of variables related to leakage from the ostomy equipment

Variables	Estimate	CI
Ostomy placed according to international guidelines (yes = 0, no = 1)	0.27	0.25, 0.32
Other diseases or conditions (no = 0, yes = 1)	0.15	0.12, 0.18
Ostomy baseplate (flat = 0, convex = 1)	0.14	0.12, 0.16
The ostomy's shape (round = 0, oval =1)	0.13	0.12, 016
Self‐care of the ostomy (yes = 0, no = 1)	0.11	0.08, 0.13
Preoperative ostomy site marking according to international guidelines (yes = 0, no = 1)	0.10	0.08. 0.12
Ostomy site marked by SCN (yes = 0, no = 1)	0.09	0.08, 0.11
Two ostomies (colo and uro) (no = 0, yes = 1)	0.09	0.08, 0.11
Gender (women = 0, men = 1)	0.07	0.06, 0.08
Ileostomy (no = 0, yes = 1)	0.04	−0.04, 0.05
IBD (inflammatory bowel disease) (no = 0, yes = 1)	0.04	−0.03, 0.06
Ostomy equipment (two piece (baseplate and pouch) = 0, One piece = 1)	0.04	0.03, 0.05
Marital (Married/cohabitant = 0, living alone = 1)	0.03	0.02, 0.03
BMI (continuous)	0.02	0.00, 0.03
Urostomy (no = 0, yes = 1)	0.02	0.00, 0.02
Time since surgery (<1 year = 1, >1 year or more = 2)	0.02	−0.01, 0.02
Reduced hand/finger function (no = 0, yes = 1)	0.01	0.00, 0.01
Preoperative information before hospitalization (no = 0, yes =1)	0.01	0.08, 0.12
Sight (normal = 0, reduced = 1)	0.00	0.00, 0.00
Education (>12 years = 0, <12 years = 1)	0.00	−0.01, 0.01
Parastomal hernia/bulge (no = 0, yes = 1)	−0.01	−0.01, 0.01
The ostomy's nipple length flush to or below skin level= 0, 0.1–2 cm over skin level = 1, 2.1–4 cm over skin level = 2, 4–6 cm over skin level = 3, or >6 cm over skin level = 4	−0.03	−0.04, 0.00
Age, years (continuous)	−0.05	−0.06, −0.01
Colostomy (no = 0, yes = 1)	−0.16	−0.18, −0.08
Knowledge and skills (continuous)	−0.17	−0.19, −0.16
Cancer (no = 0, yes = 1)	−0.20	−0.23, −0.12
Health professionals (continuous)	−0.25	−0.28, 0.22

*Note*: The model explains 31% of the variance in leakage.

Abbreviation: CI, confidence interval.

#### 4.1.10 Other factors

The results found associations between higher BMI scores and leakage (Table 2), supporting previous studies' results (Arumugam et al., 2003; Braumann et al., 2019; Bulkley et al., 2018). The results also found associations between longer time since surgery and less leakage. Likewise, an Irish study including respondents having ostomy for up to 15 years showed that about one of three had leakage challenges (Davidson, 2016). Unlike other studies, the results did not show a significant association between having a hernia and leakage (Cowin & Redmond, 2012; Krogsgaard et al., 2017) or between genders (Ratliff, 2014). In this study, only 5.6% of the patients had dermatitis, and 13.1% had other skin disorders. To determine the association between patient‐reported leakage and peristomal complications in an outpatient clinic is difficult because the SCN often only observes the skin on the day the patient has an appointment. Peristomal complications can cause leakage and vice versa. Several factors can cause peristomal complications, such as stripping effect, moisture, granulomas, skin disorders, allergy, and infections (Colwell et al., 2011; Salvadalena, 2013). One study about nursing specialists' practice in treating sore skin found that about 50% of the patients had sore skin, and in 61%, the reason was leakage from the ostomy equipment (Burch, 2014). A separate study found that sore skin and leakage were two of the eight most prominent themes (Sun et al., 2020).

### 4.2 Implications for practice

It is suggested that to explore reasons for leakage, the focus should be on individualized education in mapping the causes of leakage and the therapeutic alliance between patient and SCN. PROs should be used together with a clinical investigation conducted by an SCN, followed by discussions of the findings and suggestions for reducing leakage frequency. Education could be individualized by understanding individual educational needs based on cooperation between patient and SCN. Recommendations and theory should guide the individualized systematic follow‐up and education program to elaborate and handle leakage about bowel or urinary tract, skin, leakage, and use of ostomy equipment and accessories (Olsen et al., 2020). It should be conducted by SCNs (García‐Goñi, 2019). Planning the education program should be based on principles in the didactic relational model (Bjørndal & Lieberg, 1978).

### 4.3 Strengths and limitations

The study has some limitations. First, leakage data were self‐reported, which might be associated with measurement error. There was no data on dietary factors, such as consistency regulation interventions for faecal output, gas, urine pH, stenosis problems, or data about the degree of heavy physical workload and how physically active the patients were. Second, there was no distinction between loop ostomies and end ostomies. Third, using the length of the ostomy as a factor associated with leakage may be helpful. Still, in particular, ileostomies have strong peristaltic activity, and the length may change during the day. Fourth, the opening in the ostomy may not be on the top of the ostomy; instead, it may be near to skin level, although the ostomy has an optimal length. Both stoma length and placement of the opening may lead to leakage, and indeed, ostomy length may be an unstable factor for leakage. Fifth, parastomal skin problems are often associated with leakage, but the skin's condition around the ostomy could cause leakage and be an effect of leakage. Thus, the patient's skin condition can be both a cause and a result of leakage, so it was not included in the regression model for variables that may affect leakage, as it may be both a strength and a weakness. Finally, it should be emphasized that the study used an explorative statistical approach rather than a strict hypothesis testing approach. A strength of this study is the high participation rate (nearly 100%) and a large set of variables in the PLS‐model.

## 5 CONCLUSIONS

The leakage from ostomy equipment was associated with several diverse, changeable factors, such as the patient's knowledge and skills, relationship to healthcare providers, and self‐care with practical ostomy care. Other factors are unchangeable, such as optimal ostomy placement, the reason for surgery, and the shape of the ostomy. Still, the patient must consider those factors in daily ostomy care. Future research should investigate individualized patient education in mapping leakage and closer cooperation between patients and SCN to explore reasons for leakage.

## Acknowledgements

We thank the patients in the user panel for their help during the study.

## AUTHOR CONTRIBUTIONS

KLI, TEO, AA, JRA have made substantial contributions to conception and design, or acquisition of data, or analysis and interpretation of data; Been involved in drafting the manuscript or revising it critically for important intellectual content; Given final approval of the version to be published. Each author should have participated sufficiently in the work to take public responsibility for appropriate portions of the content; And agreed to be accountable for all aspects of the work in ensuring that questions related to the accuracy or integrity of any part of the work are appropriately investigated and resolved.

## CONFLICT OF INTEREST

The authors declare no competing interests.

## TRIAL REGISTER

ClinicalTrials.gov Registration Number: NCT03841071. Date 18. February 2019 retrospectively registered.

## Data Availability

The dataset generated during this study will not be publicly available as the patient consent and approval from the Regional Committee for Medical and Health Research Ethics prevents sharing of individual patient level data in public repositories. However, the data will be available from the corresponding author upon reasonable request.
